# Temporal increase in the incidence of anal squamous cell carcinoma in Kentucky and factors associated with adverse outcomes

**DOI:** 10.1002/cam4.5865

**Published:** 2023-03-29

**Authors:** Stephen J. O'brien, Jeremy T. Gaskins, C. Tyler Ellis, Brock A. Martin, Jaclyn Mcdowell, Dibson Dibe Gondim, Susan Galandiuk

**Affiliations:** ^1^ Price Institute of Surgical Research and Division of Colorectal Surgery, Hiram C. Polk Jr. MD Department of Surgery, University of Louisville School of Medicine Louisville Kentucky USA; ^2^ Department of Bioinformatics and Biostatistics, School of Public Health & Information Sciences University of Louisville Louisville Kentucky USA; ^3^ Department of Pathology and Laboratory Medicine University of Louisville Louisville Kentucky USA; ^4^ Markey Cancer Control Program, Kentucky Cancer Registry Lexington Kentucky USA

**Keywords:** anal squamous cell carcinoma, HPV, human papilloma virus, incidence, p16, overall survival, recurrence‐free survival

## Abstract

**Background:**

Anal squamous cell cancer (ASCC) incidence in Kentucky is increasing at an alarming rate. In 2009, the incidence surpassed the US national average (2.66 vs. 1.77/100,000 people), and currently, Kentucky ranks second by state per capita. The reasons for this rise are unclear. We hypothesize individuals with ASCC in Kentucky have some unique risk factors associated with worse outcomes.

**Methods:**

Individuals with ASCC in a population‐level state database (1995–2016), as well as those treated at two urban university‐affiliated tertiary care centers (2011–2018), were included and analyzed separately. We evaluated patient‐level factors including demographics, tobacco use, stage of disease, HIV‐status, and HPV‐type. We evaluated factors associated with treatment and survival using univariable and multivariable survival analyses.

**Results:**

There were 1698 individuals in state data and 101 in urban center data. In the urban cohort, 77% of patients were ever‐smokers. Eighty‐four percent of patients had positive HPV testing, and 58% were positive for HPV 16. Seventy‐two percent of patients were positive for p16. Neither smoking, HPV, nor p16 status were associated with disease persistence, recurrence‐free survival, or overall survival (all *p* > 0.05). Poorly controlled HIV (CD4 count <500) at time of ASCC diagnosis was associated disease persistence (*p* = 0.032). Stage III disease (adjusted HR = 5.25, *p* = 0.025) and local excision (relative to chemoradiation; aHR = 0.19, *p* = 0.017) were significantly associated with reduced recurrence‐free survival.

**Conclusions:**

The rate of ASCC in Kentucky has doubled over the last 10 years, which is outpacing anal SCC rates in the US with the most dramatic rates seen in Kentucky women. The underlying reasons for this are unclear and require further study. There may be other risk factors unique to Kentucky.

## Abstract 2

The incidence of Anal Squamous Cell carcinoma is markedly increasing in Kentucky and in the United States overall. This difference is more pronounced in women and may be due to the interplay of factors including smoking and human papilloma virus infection.

## 1 INTRODUCTION

Anal squamous cell carcinoma (ASCC) is uncommon, accounting for only 1% of all gastrointestinal cancers. The incidence, however, is increasing globally.^1^, ^2^, ^3^ In the United States, ASCC incidence and mortality rates are increasing at annual rates of 2.7% and 3.1%.^2^ In Kentucky, the rate of ASCC was below the US average in 1996, but surpassed the national average in 2005. Kentucky now has the second highest incidence of ASCC by state—Kentucky 3.05 cases/100,000 versus US 1.71/100,000 individuals in 2016.^4^

The rise of ASCC in Kentucky is perplexing but is in accordance with our clinical practice. The driving factors behind Kentucky's upward trend are unknown. Known risk factors for ASCC include (1) human papilloma virus (HPV), notably types 16 and 18, with associated precancerous anal intraepithelial neoplasia (AIN),^5^ (2) sexually transmitted diseases and promiscuity, (3) anal intercourse, (4) smoking tobacco use,^6^ (5) human immunodeficiency virus (HIV)‐infection, associated low CD4 count, and the duration of disease, and (6) immunosuppression secondary to solid organ transplantation or other reasons.^7^, ^8^ It is not known if the prevalence of all/some of these risk factors is disproportionately higher in Kentucky compared to US national figures. Additionally, some of these risk factors (e.g., smoking, HPV status, and HIV infection) are not included as variables, in US data sets examining ASCC.^2^ Furthermore, anecdotal evidence suggests that individuals who develop ASCC in Kentucky, compared with national cohorts, have lower rates of successful curative treatment with chemoradiotherapy and worse overall survival.

The aim of this study was to investigate factors associated with these temporal trends, treatment, and outcomes in a population‐based Kentucky state cancer registry. An analysis of more granular data from two urban tertiary referral centers was also performed. We hypothesize the following: (1) risk factors are discordant with national trends, and (2) there may be increased treatment failure and worse survival among those diagnosed with ASCC in Kentucky compared to US reports.

## 2 METHODS

Two separate cohorts were examined for this study; (1) The Kentucky Cancer Registry and (2) University of Louisville affiliated Cancer Centers (University of Louisville Brown Cancer Center and the Norton Cancer Institute). The latter group will be referred to the “Urban cohort” throughout the text.

### 2.1 Kentucky population‐based cohort

The Kentucky Cancer Registry (KCR) is a premier population‐based cancer registry. Since 2000, it has been a part of the National Cancer Institute Surveillance, Epidemiology, and End Results (SEER) program and the Center for Disease Control and Prevention's (CDC) National Program of Cancer Registries (NPCR).^9^ Data elements collected by KCR can be found in the Abstractor's Manual.^9^

All individual diagnosed with ASCC who reside in Kentucky between 1995 and 2016 were included in our cohort. Cases were identified by KCR using International Classification of Diseases for Oncology, 3rd Edition (ICD‐O‐3) site and histology codes for anal SCC (C21‐C21.8). The following histologic types were excluded from anal SCC: melanomas (8720–8790), sarcomas (8800–8991), mesotheliomas (9050–9055), Kaposi sarcomas (9140), and leukemias/lymphomas (9590–9992). Individual‐level factors available included the following: demographics, stage of disease, initial treatment, disease recurrence, and overall survival. Individuals were followed from the date of initial diagnosis to the date of last contact or date of death. National and Kentucky anal SCC incidence rates were identified from the SEER database and KCR.^4^, ^9^ Appalachia is a region in the eastern United States that spans from southern New York to northern Mississippi, including eastern Kentucky. It has higher rates of poverty and poor healthcare outcomes compared to other parts of the United States. Appalachian population statistics were recorded from census data.^10^

### 2.2 Urban tertiary centers cohort

Individuals treated for ASCC at two large urban tertiary centers in the same city in Kentucky between 2014 and 2019 were evaluated in a separate analysis to provide more granular data on individuals with ASCC. This convenient sample of patients are also included in our KCR cohort; however, we did not attempt to link or re‐identify these individuals. Patients with American Joint Commission on Cancer (AJCC) Stage I‐III were included for analysis. Additional individual‐level factors available that are known risk factors for ASCC were included, specifically: tobacco smoking history, Human Papilloma Virus (HPV), p16, and human immunodeficiency virus (HIV). HPV typing was performed at the Moffitt Cancer Center, University of South Florida. HPV types were classified using a hierarchical attribution into two mutually exclusive categories: (1) HPV16 positive regardless of multiple HPV infections, (2) all other HPV types, or (3) HPV negative.

Immunohistochemistry (IHC) expression of p16 was determined by using paraffin‐embedded tumor biopsies; this was performed by the Special Procedures Laboratory in the Department of Pathology, University of Louisville. Expression of p16^INK4A^ (p16 positive) is strongly correlated with HPV infection, but there is known discordance between the two.^11^ Expression of p16 is clinically relevant as it is associated with improved prognosis.^12^

Comorbidities were classified using the Charlson comorbidity index (CCI).^13^ Functional status was measured according to the Eastern Cooperative Oncology Group (ECOG) performance status.^14^ Tobacco smoking was categorized in relation to the date of diagnosis as never smoker, ex‐smoker (>6 months not smoking before diagnosis), and current smoker. An “ever‐smoker” is defined as an individual who had ever smoked prior to diagnosis and includes both current and ex‐smokers. Staging for anal cancer was according to the AJCC staging system.^15^ Details of primary and secondary treatment modalities were recorded for each patient.

### 2.3 Study outcomes

*Primary*: (1) Incidence of ASCC in Kentucky compared to US. (2) Temporal changes in individual factors associated with an ASCC diagnosis in Kentucky from 1996–2007 compared to 2008–2016.

*Secondary*: Patients were followed from the date of diagnosis to the date of last contact or the date of death. If an individual was never recorded as becoming free from disease (i.e., had clinical evidence of residual disease at follow‐up), disease persistence was recorded. Recurrence was defined a local or distant cancer recurrence following an interval‐free period from disease between initial treatment and recurrence. The number of days from the date initial diagnosis to cancer recurrence was calculated for each patient.

### 2.4 Statistical analysis

Statistical analysis was performed using SPSS v26 (IBM Corp, Armonk, NY) and R statistical software v4.2.1 (R Foundation for Statistical Computing). Categorical variables are presented as number and frequency. Continuous variables are presented as median (interquartile range). Categorical variables were compared using Fisher's exact test. Continuous variables were compared using the Mann–Whitney U‐test, unless otherwise stated. Univariable analyses between groups were performed using the Fisher's exact test and the Mann–Whitney U‐test. Recurrence‐free survival and overall survival rates were computed using Kaplan–Meier plots. Associations with categorical and continuous variables were investigated using the Cox proportional hazards model; log‐rank *p*‐values were reported as an overall effect for categorical factors with more than two levels. Multivariable Cox models were constructed using all predictors that were significant on marginal analysis, and the appropriateness of the proportional hazards assumption was confirmed using the Schoenfeld residuals global test.^16^ All statistical tests were two‐tailed, and when variables include missing values, an available case analysis is performed. Graphs were made using GraphPad Prism 7 (GraphPad Software Inc.).

### 2.5 Ethics statement

This study was approved by the Institutional Review Boards of the University of Louisville and the University of Kentucky (Kentucky Cancer Registry at the Markey Cancer Center, University of Kentucky).

## 3 RESULTS

### 3.1 Kentucky population‐based cohort

**FIGURE 1:**
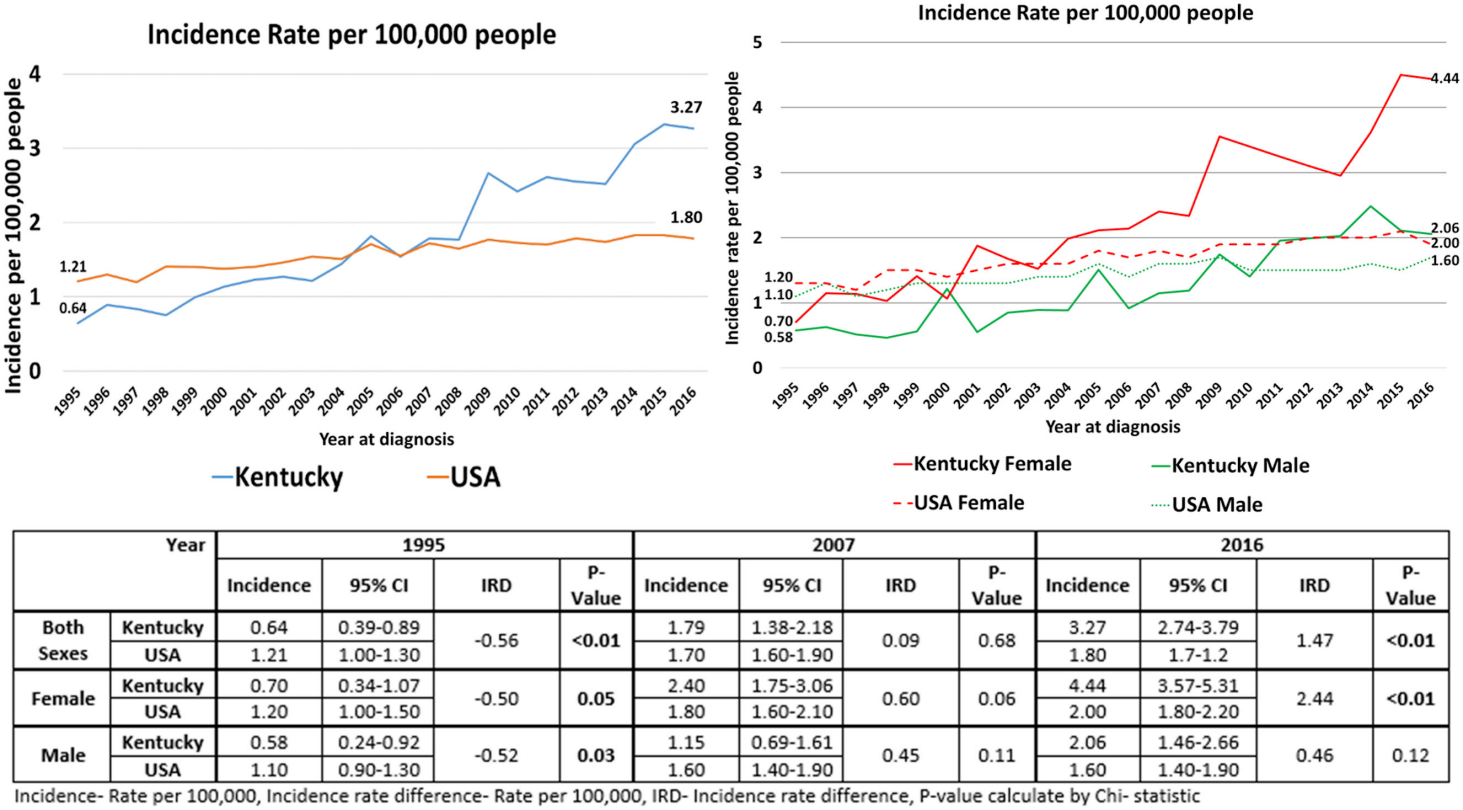
Temporal increase in the incidence of anal SCC in Kentucky and in the United States overall.

Between 1995 and 2016, 1698 individuals were diagnosed with ASCC in Kentucky. The annual incidence rose from 2.4 to 3.3/100,000 individuals during this study period, which surpassed the national average in 2005, Figure 1.^9^ A large spike in incidence was seen after 2008 in Kentucky. The major difference in incidence relative to national rates occurred with female patients in Kentucky (Incidence rate difference 2.44 × 100,000, *p* < 0.01). There was no difference in the incidence between the male patients in Kentucky compared to the US. Figure 1. There was no difference in the incidence rates between patients living in Appalachia compared to the non‐Appalachia regions in Kentucky, Figure S1.

In Kentucky, there were differences in demographics and stage of disease at diagnosis between the pre‐ and post‐2008, Table 1. Rates of ASCC in women over the entire study period were consistently higher than men, 66% versus 34%. There was an increase in the rate of tobacco smoking among both men and women diagnosed with ASCC over time, 69% versus 74%, *p* = 0.049, as well as a decrease in age at diagnosis (median 57 vs. 55, *p* = 0.026). An increasing number of individuals were insured with Medicaid, 11% to 16%, or managed care insurance, 20% to 29%. There was a significant difference in the distribution of AJCC stage between the time periods (*p* = 0.021), Table 1.

**TABLE 1:** Demographics of individuals diagnosed with Anal Squamous Cell Carcinoma in Kentucky 1995–2016. (Kentucky Cancer Registry).

Variable	1995–2007	2008–2016	Total	*p*‐value
	*N* = 638	*N* = 1060	*N* = 1698	
	*N* (%)	*N* (%)	*N* (%)	
Demographics				
Age, median (IQR)	**57 (46–69)**	**55 (47–65)**	**56 (46–67)**	**0.026**
Sex				
Male	215 (34)	366 (35)	581 (34)	0.752
Female	423 (66)	694 (65)	1117 (66)	
Race				
White	593 (93)	970 (92)	1563 (92)	0.102
African American	43 (7)	76 (7)	119 (7)	
Other	2 (0)	14 (1)	16 (1)	
Smoking				
Ever smoker^a^	**352 (69)**	**682 (74)**	**1034 (72)**	**0.049**
Non smoker	**158 (31)**	**239 (26)**	**397 (28)**	
(Missing)	**128**	**139**	**267**	
Living in Appalachia				
Yes	157 (25)	266 (25)	423 (25)	0.862
No	481 (75)	794 (75)	1275 (75)	
Insurance status				
Uninsured	**38 (6)**	**76 (7)**	**114 (7)**	**<0.001**
Medicaid	**69 (11)**	**165 (16)**	**234 (14)**	
Medicare	**267 (43)**	**394 (38)**	**661 (40)**	
Military	**11 (2)**	**21 (2)**	**32 (2)**	
Insurance, not specified	**107 (17)**	**79 (8)**	**186 (11)**	
Managed care (HMO, PPO)	**123 (20)**	**299 (29)**	**422 (26)**	
(Missing)	**23**	**26**	**49**	
AJCC stage				
Stage 0	**105 (19)**	**247 (23)**	**352 (22)**	**0.021**
Stage I	**116 (21)**	**181 (17)**	**297 (18)**	
Stage II	**238 (43)**	**402 (38)**	**640 (40)**	
Stage III	**77 (14)**	**187 (18)**	**264 (16)**	
Stage IV	**21 (4)**	**38 (4)**	**59 (4)**	
(Missing)	**81**	**5**		
Initial therapy				
No therapy	49 (8)	71 (7)	120 (7)	0.313
Local excision	274 (43)	507 (48)	781 (46)	
Abdominoperineal resection	18 (3)	23 (2)	41 (2)	
Chemoradiotherapy	263 (41)	410 (39)	673 (40)	
Chemotherapy alone	19 (3)	15 (1)	30 (2)	
Radiotherapy alone	19 (3)	34 (3)	53 (3)	
Chemoradiotherapy prior to APR (among pts receiving both CRT & APR)				
Yes	35 (63)	45 (65)	80 (64)	0.899
No	21 (37)	24 (35)	45 (36)	

*Note*: Categorical variables were compared using Fisher exact test. Continuous variables were compared using Mann–Whitney U‐test. Boldface indicates statistical significance.

Abbreviations: AJCC, American Joint commission on Cancer staging, APR, Abdominoperineal resection.

^a^ Ever‐smoker refers to a patient who is a current or ex‐smoker in the KCR cohort.

The most common initial treatment was local excision (46%), followed by chemoradiotherapy (40%) (Table 1). Among the whole cohort, 34% had disease persistence, and 6% had an observed recurrence (Table 2). In subset analysis including only those with stage I‐III disease and receiving treatment (*n* = 1201), the persistence rate was 37%. Chemoradiotherapy was associated with increased rates of disease persistence (Table S1). Overall survival increased from 1995–2007 to 2008–2016, with 76% patients estimated to be alive 3 years post‐diagnosis (Figure S2). In stage I patients, chemoradiotherapy was associated with worse overall survival compared to local excision. In stage II patients, both APR and chemoradiotherapy were associated with worse overall survival compared to local excision. There was no difference in overall survival when comparing treatment modalities in stage III patients (Table S2). However, full information on the intended treatment strategies and their rationale is not available from KCR, and their interpretation must be considered cautiously.

**TABLE 2:** Clinical outcomes for individuals diagnosed with Anal Squamous Cell Carcinoma in Kentucky 1995–2016. (Kentucky Cancer Registry).

Variable	1995–2007	2008–2016	All patients	*p*‐value
	*N* = 638	*N* = 1060	*N* = 1698	
	*N* (%)	*N* (%)	*N* (%)	
Outcome				
Disease persistence after initial therapy				
Yes	205 (32)	370 (35)	575 (34)	0.245
No	433 (68)	690 (65)	1123 (66)	
Disease persistence after initial therapy (treated, stages 1–3)				
Yes	127 (32)	251 (35)	378 (34)	0.235
No	273 (68)	458 (65)	731 (66)	
Tumor recurrence (among disease free)*				
Yes	**54 (12)**	**55 (8)**	**109 (10)**	**0.017**
No	**379 (88)**	**635 (92)**	**1014 (90)**	
Time to recurrence (among disease free)				
3 year rate	90% (87–93)	92% (90–94)	91% (90–93)	**Log‐rank** *p* = 0.090
Recurrence‐Free Survival (among disease free)				
3 year Rate	**78% (75–82)**	**84% (81–87)**	**82% (79–84)**	**Log‐rank** *p* **= 0.002**
Vital status				
Dead	**393 (62)**	**301 (28)**	**694 (41)**	**<0.001**
Alive	**245 (38)**	**759 (72)**	**1004 (59)**	
Overall survival				
3 year Rate	**73% (70–77)**	**77% (75–80)**	**76% (73–78)**	**Log‐rank** *p* **= 0.001**
Length of follow‐up (months), median (IQR)	**117 (32–166)**	**45 (22–77)**	**55 (25–105)**	**Log‐rank** *p* **< 0.001**

*Note*: Categorical variables were compared using Fisher's exact test. Survival outcomes analyzed using Kaplan–Meier curves, reporting the rates at the 3‐year mark, and compared using the log‐rank test. *Recurrence in KCR is defined as time from disease free after initial therapy to new tumor recurrence along among those who are disease free. Time to recurrence is defined using death as a censoring event. Analysis of length of follow‐up uses the reverse Kaplan–Meier method (loss to follow‐up is the event of interest; death is a censoring event). Boldface indicates statistical significance.

When we compared pre‐ and post‐Medicaid expansion (1995–2012 vs 2013–2016) in the KCR data set, there was an increase in the percentage of individuals insured by Medicaid (pre‐expansion 138/1126, 12%, to post‐expansion 96/523, 18%, Fisher *p* = 0.001).

### 3.2 Urban tertiary centers cohort

Between 2011 and 2018, 101 individuals were treated for stage I‐III ASCC at two urban centers. Tables 3, 4, 5 represent data from the Urban cohort of patients. The median age of diagnosis was 57 years, and the majority of patients were female. Eighty‐four percent of patients were positive for any HPV type, with HPV‐16 being the most common (70% of HPV patients). The distribution of the HPV types is shown in Table S3. Seventy‐two percent of all individuals had positive p16 immunohistochemistry. Concordance between the presence of any HPV type and p16 positivity was 81%. Nineteen (19%) were positive for HIV with the median duration of disease of 8 years, Table 3. This median value was used to dichotomize HIV duration into short and long disease duration.

**TABLE 3:** Factors associated with disease persistence following initial treatment in the urban cohort.

Variable	No disease persistence	Disease persistence	All patients	*p*‐value
	*N* = 87	*N* = 13	*N* = 101^a^	
	*N* (%)	*N* (%)	*N* (%)	
Demographics				
Age median (IQR)	58 (51–66)	50 (38–57)	57 (50–66)	0.051
Gender				
Female	53 (61)	6 (46)	60 (59)	0.372
Male	34 (39)	7 (54)	41 (4)	
Race				
White	71 (83)	10 (77)	82 (82)	0.734
African American	14 (16)	3 (23)	17 (17)	
Other	1 (1)	0 (0)	1 (1)	
(Missing)	1		1	
Smoking				
Current Smoker	42 (49)	5 (38)	48 (48)	0.744
Ex‐smoker	25 (29)	4 (31)	29 (29)	
Never	19 (22)	4 (31)	23 (23)	
(Missing)	1	0	1	
Charlson Comorbidity status				0.161
0	9 (10)	4 (31)	13 (13)	
1–2	35 (40)	4 (31)	39 (39)	
>3	43 (49)	5 (38)	49 (49)	
BMI—kg/m^2^, Median (IQR)	26.3 (23.3–32.4)	29.0 (21.7–31.7)	26.3 (23.2–32.0)	0.853
Human Papilloma Virus				
Yes	57 (84)	10 (83)	67 (84)	1.000
No	11 (16)	2 (17)	13 (16)	
(Missing)	19	1	21	
Human Papilloma Virus				
HPV including serotype 16	41 (61)	5 (42)	46 (58)	0.344
HPV without serotype 16	15 (22)	5 (42)	20 (25)	
Negative	11 (16)	2 (17)	13 (16)	
(Missing)	19	1	21	
P16 expression				
Negative	**6 (10)**	**4 (33)**	**10 (14)**	**0.024**
Equivocal	**7 (11)**	**3 (25)**	**10 (14)**	
Positive	**49 (79)**	**5 (42)**	**54 (72)**	
(Missing)	**25**	**1**	**27**	
Human Immunodeficiency Virus				
Yes	14 (16)	5 (38)	19 (19)	0.069
No	73 (84)	8 (62)	82 (81)	
HIV duration of disease^b^				
<8 years	7 (50)	2 (40)	9 (47)	1.000
>8 years	7 (50)	3 (60)	10 (53)	
CD4+ Load at diagnosis, cells/mL, median (IQR)^b^	**366 (217–861)**	**221 (67–289)**	**303 (160–608)**	**0.046**
(Missing)	**1**	**0**	**1**	
HIV and disease control				**0.032**
No HIV	**73 (85)**	**8 (61)**	**82 (82)**	
Well controlled HIV (CD4 > 500)	**5 (6)**	**0 (0)**	**5 (5)**	
Poorly controlled HIV (CD4 < 500)	**8 (9)**	**5 (39)**	**13 (13)**	
(Missing)	**1**	**0**	**1**	
ECOG status				1.000
0, 1	78 (96)	11 (92)	90 (96)	
2, 3, 4	3 (4)	1 (8)	4 (4)	
(Missing)	6	1	7	
AJCC Stage				
Stage I	22 (25)	2 (15)	25 (25)	0.086
Stage II	34 (49)	2 (15)	36 (36)	
Stage III	31 (36)	9 (69)	40 (40)	
Therapy				0.850
Local excision	9 (10)	2 (15)	12 (12)	
Abdominoperineal resection	3 (3)	0 (0)	3 (3)	
Chemoradiotherapy	72 (83)	11 (85)	83 (82)	
Chemotherapy	3 (3)	0 (0)	3 (3)	

*Note*: Categorical variables were compared using Fisher's exact test. Continuous variables were compared using Mann–Whitney U‐test. Boldface indicates statistical significance.

Abbreviations: AJCC, American Joint Commission on Cancer; HIV, Human Immunodeficiency Virus; HPV, Human Papilloma Virus; IQR, Interquartile Range,

^a^ *n* = 1 patient without information on disease persistence.

^b^ HIV positive only.

**TABLE 4:** Univariable and multivariable Cox proportional hazards regression analysis for risk factors associated with reduced recurrence‐free survival in the urban cohort.

Variable (*N* = 101, 39 events)	Univariable analysis			Multivariable analysis		
	Hazard ratio	95% CI	*p*‐value	Hazard ratio	95% CI	*p*‐value
Age	0.99	0.97–1.02	0.568			
Gender—Male	**2.03**	**1.07–3.84**	**0.030**	1.71	0.70–4.15	0.237
Race			LR = 0.840			
White	1					
Black	0.76	0.29–2.02	0.582			
Other	0.00	xxx	xxx			
Smoking			LR = 0.573			
Never	1					
Ex‐smoker	1.51	0.63–3.61	0.354			
Current Smoker	1.10	0.47–2.58	0.821			
Charlson Comorbidity status			LR = 0.169			
0	1					
1–2	0.61	0.21–1.79	0.368			
>3	1.23	0.47–3.27	0.670			
BMI	0.99	0.95–1.04	0.742			
Human Papilloma Virus‐positive	1.36	0.50–3.67	0.545			
HPV			LR = 0.513			
Negative	1					
HPV other than 16	1.80	0.59–5.49	0.303			
HPV 16	1.22	0.44–3.42	0.704			
P16 status			LR = 0.051			
Negative	1					
Equivocal^a^	1.10	0.34–3.51	0.875			
Positive	0.43	0.16–1.18	0.101			
Human Immunodeficiency Virus	1.71	0.77–3.77	0.186			
HIV			**LR = 0.006**			
Negative	**1**			1		
<8 years	**0.66**	**0.16–2.78**	**0.573**	0.28	0.06–1.37	0.117
>8 years	**3.75**	**1.51–9.30**	**0.004**	1.47	0.49–4.44	0.496
HIV and disease control			LR = 0.209			
No HIV	1					
Well controlled HIV (CD4 > 500)	1.18	0.90–5.46	0.084			
Poorly controlled HIV (CD4 < 500)	2.21	0.28–4.93	0.825			
ECOG status					0.68–11.25	0.153
0, 1	**1**			1		
2, 3, 4	**6.51**	**1.86–22.71**	**0.003**	2.78		
AJCC Stage			**LR <0.001**			
Stage I	**1**			**1**		
Stage II	**0.58**	**0.21–1.63**	**0.301**	**0.77**	**0.18–3.21**	**0.716**
Stage III	**3.27**	**1.43–7.51**	**0.005**	**5.25**	**1.23–22.44**	**0.025**
Therapy			**LR = 0.038**			
Local Excision	**1**			**1**		
Abdominoperineal resection	**2.02**	**0.42–9.80**	**0.382**	**2.24**	**0.18–28.00**	**0.533**
Chemoradiotherapy	**0.47**	**0.20–1.07**	**0.073**	**0.19**	**0.04–0.74**	**0.017**
Chemotherapy	**0.00**	**xxx**	**xxx**	**0.00**	**xxx**	**xxx**

*Note*: Boldface indicates statistical significance; LR provides the *p*‐value for the categorical predictors using the log‐rank test; xxx indicates confidence interval and *p*‐values cannot be estimated due to no events within subgroup.

Abbreviations: AJCC, American Joint Commission on Cancer; ECOG, Eastern Cooperative Oncology Group; HPV, Human Papilloma Virus.

^a^ Equivocal—Following review by two board certified pathologists, the immunohistochemistry slides did not meet the criteria for a positive or negative call.

**TABLE 5:** Univariable and multivariable Cox proportional hazards regression analysis for risk factors associated with reduced overall survival.

Variable (*N* = 101, 27 events)	Univariable analysis			Multivariable analysis		
	HR	95% CI	*p*‐value	HR	95% CI	*p*‐value
Age	1.00	0.97–1.03	0.878			
Gender‐Male	2.03	0.94–4.38	0.073			
Race			LR = 0.751			
White	1					
Black	1.42	0.56–3.60	0.460			
Other	0.00	xxx	xxx			
Smoking			LR = 0.643			
Never	1					
Ex‐smoker	0.71	0.26–1.89	0.49			
Current smoker	0.65	0.25–1.66	0.37			
Charlson comorbidity status			LR = 0.147			
0	1					
1–2	0.43	0.08–2.35	0.328			
>3	1.19	0.28–5.12	0.813			
BMI	0.98	0.93–1.04	0.504			
Human Papilloma Virus	1.09	0.32–3.77	0.891			
HPV			LR = 0.611			
Negative	1					
HPV other than 16	1.55	0.39–6.21	0.54			
HPV 16	0.97	0.27–3.50	0.97			
P16 status			LR = 0.088			
Negative	1					
Equivocal	1.71	0.41–7.18	0.465			
Positive	0.55	0.15–1.98	0.357			
Human Immunodeficiency Virus	1.12	0.38–3.28	0.842			
HIV			LR = 0.108			
Negative	1					
<8 years	0.00	xxx	xxx			
>8 years	2.23	0.76–6.55	0.143			
HIV and disease control			LR = 0.203			
No HIV	1					
Well controlled HIV (CD4 > 500)	0.00	xxx	xxx			
Poorly controlled HIV (CD4 < 500)	1.99	0.67–5.94	0.216			
ECOG status						
0, 1	**1**			**1**		
2, 3, 4	**8.37**	**2.36–29.69**	**0.001**	**4.76**	**1.27–17.77**	**0.020**
AJCC Stage			**LR <0.001**			
Stage I	**1**			**1**		
Stage II	**0.54**	**0.12–2.47**	**0.430**	**1.21**	**0.11–13.28**	**0.878**
Stage III	**4.29**	**1.44–12.78**	**0.009**	**14.86**	**1.28–172.88**	**0.031**
Disease persistence	**6.68**	**2.75–16.22**	**<0.001**			
Therapy			**LR = 0.013**			
Local Excision	**1**			**1**		
Abdominoperineal resection	**5.35**	**0.95–30.18**	**0.057**	**4.01**	**0.18–91.35**	**0.384**
Chemoradiotherapy	**0.71**	**0.24–2.09**	**0.529**	**0.13**	**0.01–1.38**	**0.090**
Chemotherapy	**0.00**	**xxx**	**xxx**	**0.00**	**xxx**	**xxx**

*Note*: Boldface indicates statistical significance; LR provides the *p*‐value for the categorical predictors using the log‐rank test; xxx indicates confidence interval and *p*‐values cannot be estimated due to no events within subgroup.

Abbreviations: AJCC, American Joint Commission on Cancer; ECOG, Eastern Cooperative Oncology Group; HPV, Human Papilloma Virus.

Disease persistence (*n* = 13/100) was not associated with HPV‐ or HPV‐16 status. Interestingly, p16 negativity was associated with greater disease persistence (33% vs. 10%, *p* = 0.024). HIV with a low CD4 count at ASCC diagnosis (CD4 < 500) was associated with higher rates of disease persistence when compared to HIV‐negative or HIV‐positive patients with CD4 > 500 (9% vs. 39%, *p* = 0.032). There were no differences in disease persistence rates by tobacco smoking or treatment type. Disease persistence tended to be more likely for Stage III patients, although this did not reach statistical significance (*p* = 0.086) (Table 3).

Similarity between the urban cohort and KCR was evaluated (Table S4), after restricting the KCR to include only those patients treated for stage I‐III disease. There was a similar age, sex, and smoking status distribution between the cohorts. There was a higher frequency of African American patients in the urban cohort, but there is a higher proportion of African Americans living in county surrounding Louisville compared to the overall state.^17^ Relative the KCR, patients in the urban cohort had higher stage of disease and were more likely to receive chemoradiation (instead of local excision) as the initial therapy.

There were 26 events of disease recurrence and 27 death events in the urban cohort (*n* = 101). For recurrence‐free survival (RFS), there were 39 events observed, and 1‐year and 3‐year RFS rates were estimated to be 87% (95%CI: 80–94) and 63% (53–74) (Figure S3). On univariable analysis, male gender (HR = 2.03, 95% CI: 1.07–3.84, *p* = 0.030), long‐term HIV disease (HR = 3.75, 95% CI 1.51–9.30, *p* = 0.004), poor ECOG status (HR = 6.51, 1.86–22.71, *p* = 0.003), and AJCC stage III (HR = 3.27, 1.43–7.51, *p* = 0.005) were associated with worse RCF. On multivariate analysis, chemoradiotherapy was associated with improved RFS and Stage III disease with worse RFS, Table 4.

Overall survival at one‐year post‐diagnosis is found to be 92% (95%CI: 87–98) and 74% (65–84) at 3 years (Figure S4). On univariable analysis, ECOG 2–4 status (HR = 8.37, 95% CI: 2.36–29.69, *p* = 0.001) and AJCC stage III (HR = 4.29, 1.44–12.78, *p* = 0.009) were associated with worse overall survival. On multivariable analysis, both of these variables remained significant risk factors for overall survival. Neither smoking nor HPV status were associated with overall survival, Table 5.

## 4 DISCUSSION

Rates of ASCC have increased over time in Kentucky. This state now has the second highest incidence in the US. Demographic changes that occurred over our study period include Medicaid expansion and increasing tobacco smoking rates among individuals diagnosed with ASCC in Kentucky. In general, rates of tobacco use in Kentucky have remained higher than the national average, with one of the highest smoking rates in the country with nearly 25% of adults reporting smoking at least 100 cigarettes in their lifetime.^18^ Unlike national statistics demonstrating that national smoking prevalence is decreasing, the prevalence of smoking in the KCR registry had increased albeit marginally.^18^ We postulated that access to care, vis‐à‐vis Medicaid expansion, which started in 2013, would be responsible for part of the increased incidence in ASCC in Kentucky, similar to breast cancer.^19^ There was a small shift in the number of cases diagnosed at an early stage, but not enough to suggest that lead‐time bias was the driving factor for the temporal increased incidence.

Increased rates of ASCC are presumed to be the result of changing sex practices and screening in the HIV‐population, particularly men who have sex with men (MSM). There is no evidence to suggest this is significantly contributing to the rising incidence in Kentucky. While ASCC is more common in the HIV‐positive population, HIV is less common in Kentucky compared to other states in the US. The HIV prevalence rate in Kentucky is 196 cases/100,000 individuals, which ranks 18th lowest by state. Similarly, rates of new HIV infection Kentucky have remained steady at approximately 8 cases/100,000 individuals with a male to female ratio of 4:1.^20^ Lastly, only 18% of our urban cohort were HIV‐positive at the time of their ASCC diagnosis.

The median age at diagnosis of ASCC was 57 years in the urban cohort and 56 in KCR. These results are similar to national trends. Deshmukh et al. found the highest increases in ASCC incidence and mortality in those over the age of 50.^2^

We did find rates of new cases of ASCC in females are outpacing males, Figure 1. Nationally, the incidence is 0.8 percentile points higher in women compared to men (2.9% vs. 2.1%).^21^ Regionally, women in the Midwest have one of the larger increases at 3.6%.^19^ We did not find a difference in the incidence of anal SCC between Appalachia and the rest of Kentucky. Additionally, there was no difference in the clinical outcomes between Appalachia and the rest of Kentucky, which is similar to previous study findings.^22^ Conversely, another HPV‐associated cancer, cervical cancer, has been decreasing over time in Kentucky.^23^

In our urban center cohort, 84% and 72% of individuals with ASCC had HPV infection and p16 positivity. Other studies have reported >90% of ACCC cases are associated with HPV‐positive specimens.^10^, ^24^ HPV16 was the most frequent type detected in HPV‐positive specimens, which is coordinate with other studies.^24^ In general, HPV and p16 positivity are associated with improved recurrence‐free survival and overall survival.^25^ For our urban cohort, p16 positivity was tended to associated with improved recurrence‐free and overall survival, although these effects were not statistically significant. Additionally, p16 positivity was associated with treatment success, when examining disease persistence (Table 3). This discrepancy is likely due to the small sample size of this cohort. Additionally, some of the p16 immunohistochemistry slides were called as “equivocal.” This is a representation of the reality of using clinical samples for the study. HPV infection is now a reportable variable in US cancer registries, so further information about this may be apparent with further years of follow‐up.

After initial treatment for ASCC, prior studies indicate that approximately 25% of individuals can expect to have disease persistence or recurrence.^8^ Disease persistence and disease recurrence were (1) 34% and 10% in the Kentucky population cohort and (2) 13% and 30% in the urban cohort. The rates of persistence and recurrence may be different in the KCR due to the registry coding of these variables.

Our goal was to shed light on the changing trends seen in clinical practice. We used available data and factors available, which are inherently limited. Before 2018, HPV status was not reported to KCR. The urban cohort was used to augment this but is not without its own limitations. The relatively small cohort limits the interpretation and generalizability of our findings. A number of the analyses were limited by sample size and could be subject to a type 2 error. Demographic factors such as household income^26^ and sexual practices were unavailable and have been associated with worse outcomes. Tumor markers—PD‐L1 and Ki‐67 expression—have prognostic implications but were not evaluated for this study.^27^

There are other limitations to consider in this study. As with any population or registry database, there are inherent limitations with the individual capturing the data and with information available from an individual patient's medical records. Although we postulate that HPV infection is a factor associated with increasing incidence in Kentucky and the US, but there is a paucity of national data of HPV infection in the US.

In sum, rates of ASCC in Kentucky are outpacing national trends and the driving factors remain unclear. High rates of tobacco smoking and increased access to care are associated with this trend. Clinical outcomes may be negatively affected by lower rates of HPV and p16 in ASCC cases in Kentucky. Future work will investigate additional lifestyle‐factors and genetic blueprints with the aim of uncovering novel factors associated with the increased incidence and worse clinical outcomes for individuals with ASCC especially in Kentucky.

## AUTHOR CONTRIBUTIONS

**Stephen J. O'brien:** Conceptualization (equal); data curation (equal); formal analysis (equal); funding acquisition (equal); methodology (equal); project administration (equal); resources (equal); software (equal); writing – original draft (equal); writing – review and editing (equal). **Jeremy Gaskins:** Data curation (equal); formal analysis (equal); investigation (equal); methodology (equal); software (equal); validation (equal); writing – review and editing (equal). **Clayton Tyler Ellis:** Formal analysis (equal); funding acquisition (equal); investigation (equal); methodology (equal); resources (equal); supervision (equal); writing – original draft (equal); writing – review and editing (equal). **Brock Martin:** Formal analysis (equal); investigation (equal); methodology (equal); project administration (equal); resources (equal); software (equal); supervision (equal); writing – original draft (equal); writing – review and editing (equal). **Jaclyn K McDowell:** Conceptualization (equal); data curation (equal); formal analysis (equal); methodology (equal); resources (equal); supervision (equal); validation (equal); writing – review and editing (equal). **Dibson Gondim:** Conceptualization (equal); data curation (equal); formal analysis (equal); methodology (equal); project administration (equal); resources (equal); supervision (equal); visualization (equal); writing – original draft (equal); writing – review and editing (equal). **Susan Galandiuk:** Conceptualization (equal); data curation (equal); funding acquisition (equal); investigation (equal); methodology (equal); project administration (equal); resources (equal); software (equal); supervision (equal); validation (equal); writing – original draft (equal); writing – review and editing (equal).

## FUNDING INFORMATION

This study was supported by the Transdisciplinary/Multidisciplinary Collaboration Research Planning Grant (TCRPG) from the University of Louisville and Price Trust. Presented in part at the American Society of Colon and Rectal Surgeons Annual Scientific Meeting Tampa, Florida 2nd May 2022.

## CONFLICT OF INTEREST STATEMENT

The authors declare no conflict of interest.

## Supporting information


Figure S1.

Figure S2.

Figure S3.

Figure S4.

Table S1–S4

## Data Availability

Not applicable.
