# Supplementary figures and images for: Temporal increase in the incidence of anal squamous cell carcinoma in Kentucky and factors associated with adverse outcomes
Source: Cancer Med. 2023 Mar 29;12(10):11462–74. doi: 10.1002/cam4.5865 (PMC10242335; doi:10.1002/cam4.5865)

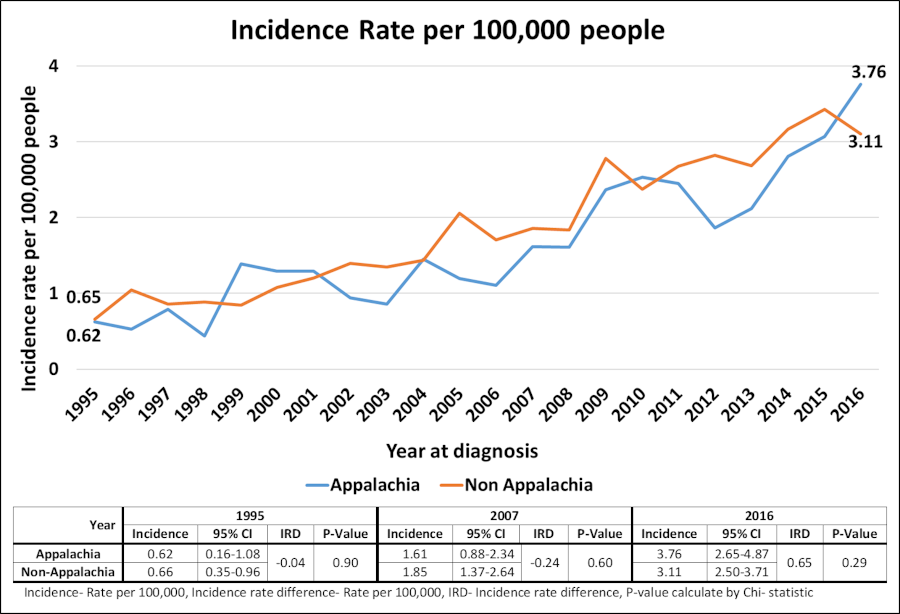

Supplement: Supplementary file 1 — Figure S1. [file CAM4-12-11462-s005.tif]

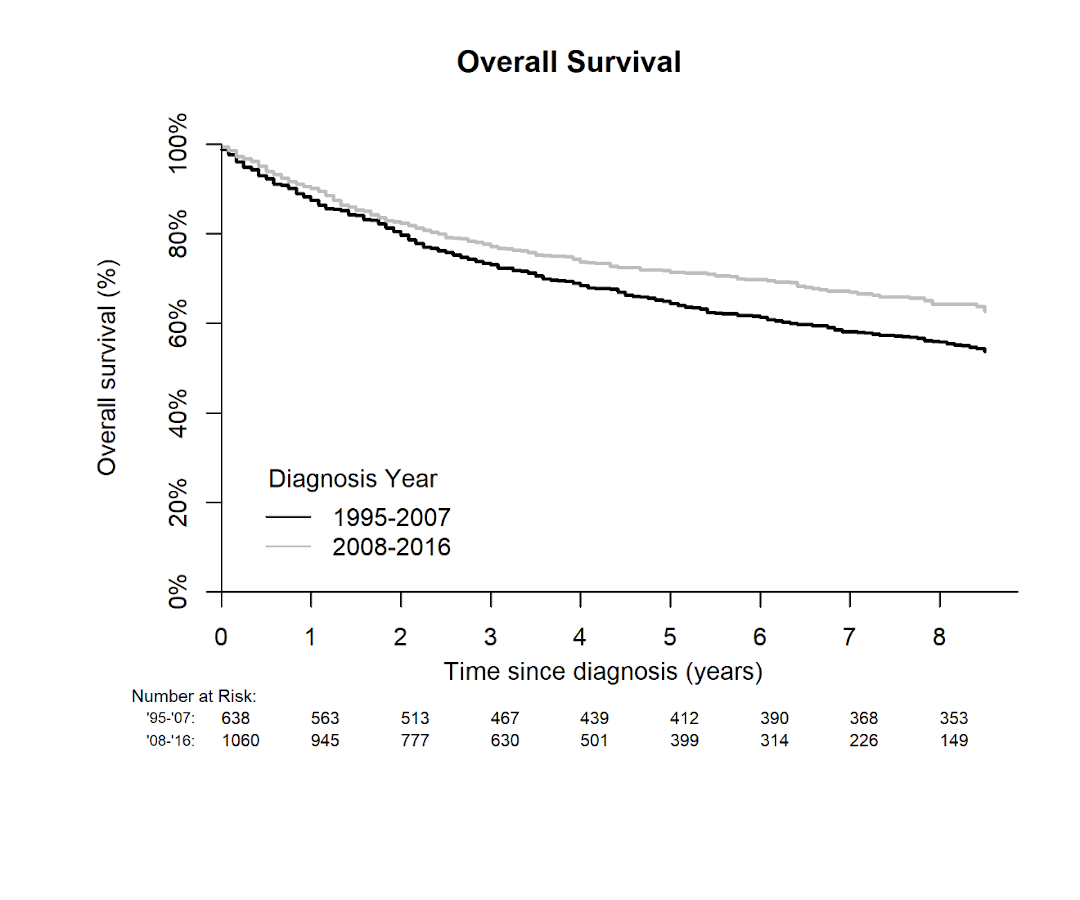

Supplement: Supplementary file 2 — Figure S2. [file CAM4-12-11462-s002.tif]

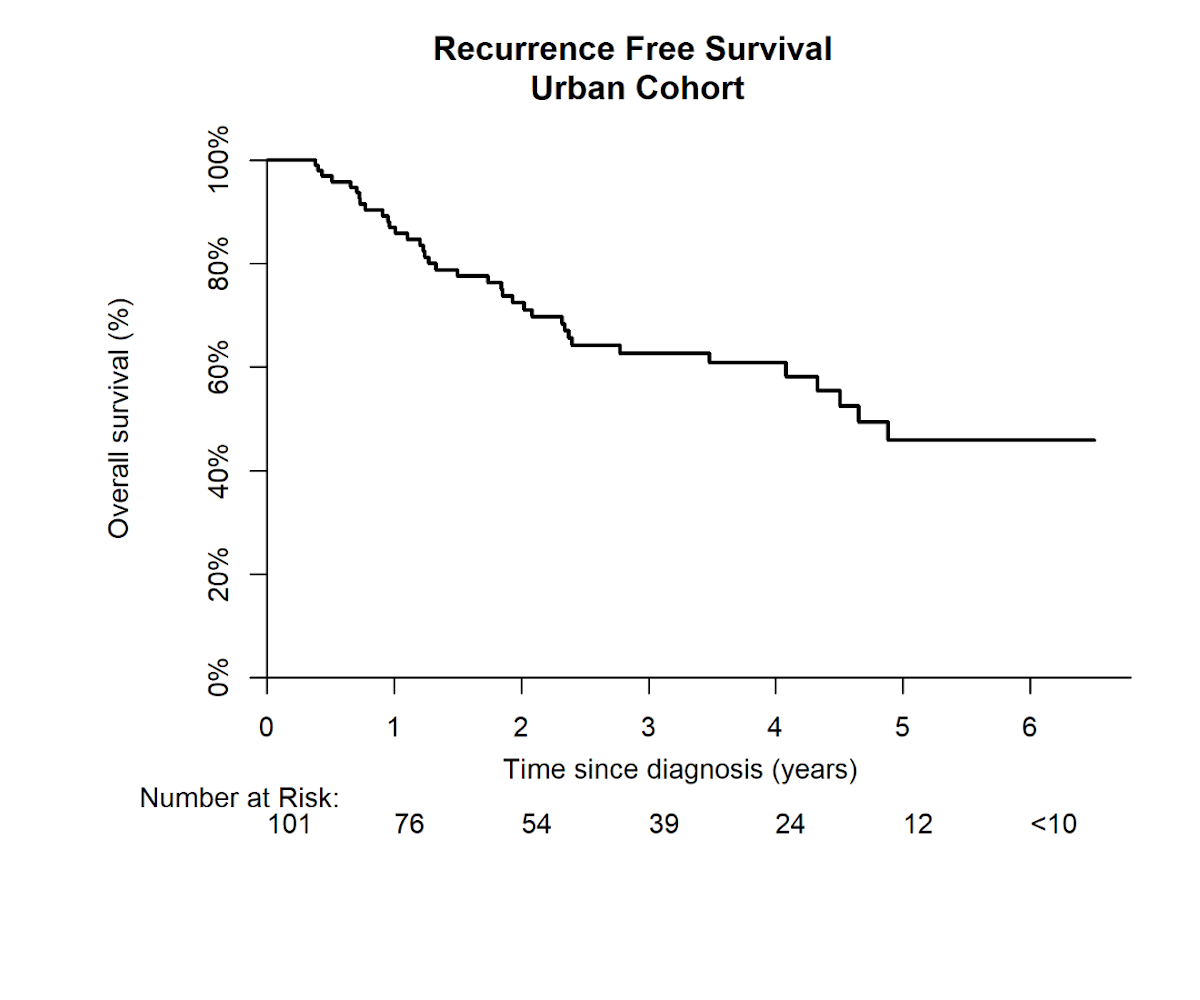

Supplement: Supplementary file 3 — Figure S3. [file CAM4-12-11462-s001.tif]

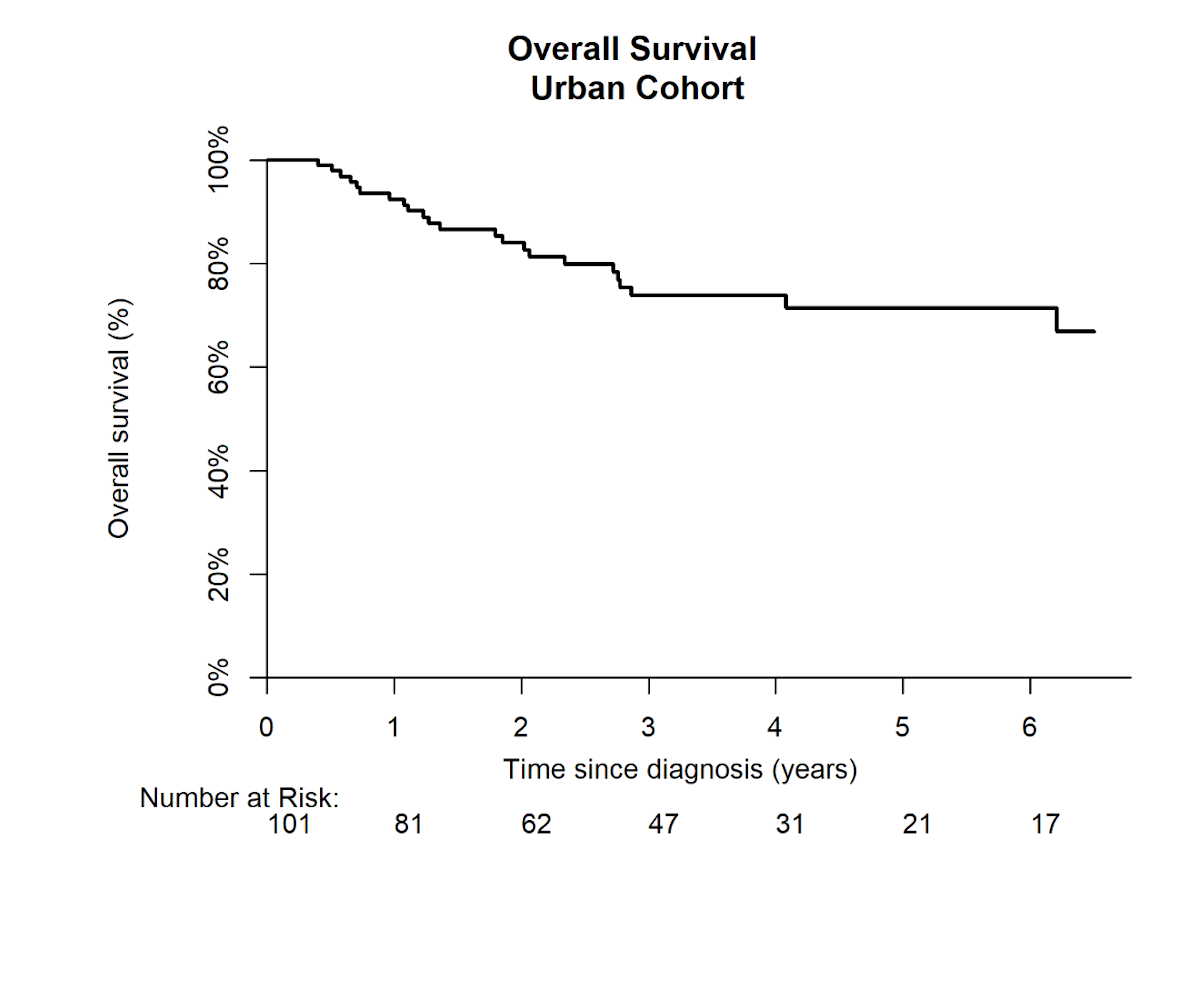

Supplement: Supplementary file 4 — Figure S4. [file CAM4-12-11462-s004.tif]
