# Supplementary material for: Temporal increase in the incidence of anal squamous cell carcinoma in Kentucky and factors associated with adverse outcomes
Source: Cancer Med. 2023 Mar 29;12(10):11462–74. doi: 10.1002/cam4.5865 (PMC10242335; doi:10.1002/cam4.5865)
Supplement: Supplementary file 5 — Table S1–S4 [file CAM4-12-11462-s003.docx]

**Supplementary Table 1. Differences in disease persistence rates between treatment strategies**

| **Variable** | **No disease persistence**  **N=731**  **N(%)** | **Disease Persistence**  **N=378**  **N(%)** | **Total Patients**  **N=1109**  **N(%)** | **P-value** | **Significant pairwise comparisons** |
| --- | --- | --- | --- | --- | --- |
| **Stage 1**  **Local Excision**  **Abdomino-perineal resection**  **Chemoradiotherapy** | 171 (74)  4 (2)  55 (24) | 32 (58)  0 (0)  23 (42) | 203 (71)  4 (1)  78 (27) | 0.025 | LE vs ChemoRT  P=0.012 |
| **Stage 2**  **Local Excision**  **Abdomino-perineal resection**  **Chemoradiotherapy** | 129 (35)  14 (4)  225 (61) | 53 (26)  1 (1)  145 (73) | 182 (32)  15 (3)  370 (65) | 0.003 | APR vs ChemoRT  P=0.012  ChemoRT vs LE p=0.023 |
| **Stage 3**  **Local Excision**  **Abdomino-perineal resection**  **Chemoradiotherapy** | 21 (16)  15 (11)  97 (73) | 13 (10)  5 (4)  106 (86) | 34 (13)  20 (8)  203 (79) | 0.031 | APR vs ChemoRT p=0.033 |
| **Stages 1-3 Combined**  **Local Excision**  **Abdomino-perineal resection**  **Chemoradiotherapy** | 321 (44)  33 (4)  377 (52) | 98 (26)  6 (2)  274 (72) | 419 (38)  39 (4)  651 (59) | <0.001 | LE vs ChemoRT  P<0.001  APR vs ChemoRT  P<0.001 |

*Chemoradiotherapy includes chemotherapy alone and radiotherapy alone. Patients receiving no treatment and those in Stage 0 and 4 are excluded from this analysis. LE-local excision, ChemoRT- chemoradiotherapy, APR- Abdominoperineal resection

**Supplementary table 2. Univariable Cox proportional hazards regression analysis for risk factors associated with reduced overall survival in the Kentucky Cancer Registry**

| **Variable** | **Log-Rank P-value** | **Hazard Ratio** | **95% CI** | **P-value** |
| --- | --- | --- | --- | --- |
| **Stage I**  **Local excision**  **Abdomino-perineal resection**  **Chemoradiotherapy** | 0.006 | 1  1.68  2.08 | 0.41-6.94  1.31-3.30 | 0.473  0.002 |
| **Stage II**  **Local excision**  **Abdomino-perineal resection**  **Chemoradiotherapy** | < 0.001 | 1  3.15  1.51 | 1.61-6.17  1.12-2.04 | <0.001  0.006 |
| **Stage III**  **Local excision**  **Abdomino-perineal resection**  **Chemoradiotherapy** | 0.103 | 1  1.57  0.89 | 0.82-3.01  0.57-1.43 | 0.170  0.649 |
| **Stages 1-3 Combined**  **Local excision**  **Abdomino-perineal resection**  **Chemoradiotherapy** | < 0.001 | 1  3.52  1.88 | 2.34-5.50  1.53-2.31 | <0.001  <0.001 |

**Supplementary Table 3. Distribution of Human Papilloma (HPV) types in the Urban Cohort**

| HPV Serotype | Frequency  N (%)* |
| --- | --- |
| 6 | 3 (5) |
| 11 | 6 (9) |
| 16 | 46 (70) |
| 18 | 2 (3) |
| 31 | 3 (5) |
| 33 | 4 (6) |
| 35 | 2 (3) |
| 39 | 2 (3) |
| 44 | 1 (2) |
| 52 | 3 (5) |
| 54 | 1 (2) |
| 68 | 1 (2) |

*n=66 patients with HPV. N’s do not sum to 66 because some patients have multiple HPV serotypes; 55 (83%) patients are positive for a single type; 9 (14%) for two types; 1 (2%) for 3 types; and 1 (2%) for five types.

**Supplementary Table 4. Differences in Demographics between the Urban Cancer Centers and the Kentucky Cancer Registry**

| **Variable** | **Urban Cancer Centers**  **N=101**  **N(%)** | **KCR***  **N=1109**  **N(%)** | **P-value** |
| --- | --- | --- | --- |
| ***Demographics*** |  |  |  |
| **Age, median (IQR)** | 57 (50-66) | 57 (49-67) | 0.975 |
| **Sex**  **Male**  **Female** | 41 (41)  60 (59) | 344 (31)  765 (69) | 0.057 |
| **Race**  **White**  **African American**  **Other** | **82 (81)**  **17 (17)**  **1 (1)** | **1039 (94)**  **67 (6)**  **3 (0)** | **<0.001** |
| **Smoking**  **Ever Smoker****  **Non smoker** | 77 (77)  23 (23) | **706 (72)**  **269 (28)** | 0.348 |
| **AJCC Stage**  **Stage I**  **Stage II**  **Stage III** | **25 (25)**  **36 (36)**  **40 (40)** | **285 (26)**  **567 (51)**  **257 (23)** | **0.001** |
| **Therapy**  **Local Excision**  **Abdominoperineal Resection**  **Chemoradiotherapy**  **Chemotherapy** | **12 (12)**  **3 (3)**  **83 (82)**  **3 (3)** | **419 (38)**  **39 (4)**  **634 (57)**  **17 (2)** | **< 0.001** |
| **Disease Persistence**  **Yes**  **No** | **13 (13)**  **87 (87)** | **378 (34)**  **731 (66)** | **< 0.001** |
| **Overall Survival**  **3yr Rate** | 74% (65-84) | 75% (72-78) | Log-rank p=0.985 |

*KCR cohort restricted to patients with Stage I-III disease and receiving treatment to match the inclusion criteria for the urban cohort.

**Former smokers in urban cohort are grouped with smokers to match Smoking status definition in KCR.
